# Thigh muscle segmentation of chemical shift encoding-based water-fat magnetic resonance images: The reference database MyoSegmenTUM

**DOI:** 10.1371/journal.pone.0198200

**Published:** 2018-06-07

**Authors:** Sarah Schlaeger, Friedemann Freitag, Elisabeth Klupp, Michael Dieckmeyer, Dominik Weidlich, Stephanie Inhuber, Marcus Deschauer, Benedikt Schoser, Sarah Bublitz, Federica Montagnese, Claus Zimmer, Ernst J. Rummeny, Dimitrios C. Karampinos, Jan S. Kirschke, Thomas Baum

**Affiliations:** 1 Department of Neuroradiology, Klinikum rechts der Isar der Technischen Universität München, Munich, Germany; 2 Department of Radiology, Klinikum rechts der Isar der Technischen Universität München, Munich, Germany; 3 Department of Sports Science, Technische Universität München, Munich, Germany; 4 Department of Neurology, Klinikum rechts der Isar der Technische Universität München, Munich, Germany; 5 Friedrich-Baur-Institut an der Neurologischen Klinik, Klinikum der Universität München der Ludwig-Maximilians-Universität, Munich, Germany; Linköping University, SWEDEN

## Abstract

Magnetic resonance imaging (MRI) can non-invasively assess muscle anatomy, exercise effects and pathologies with different underlying causes such as neuromuscular diseases (NMD). Quantitative MRI including fat fraction mapping using chemical shift encoding-based water-fat MRI has emerged for reliable determination of muscle volume and fat composition. The data analysis of water-fat images requires segmentation of the different muscles which has been mainly performed manually in the past and is a very time consuming process, currently limiting the clinical applicability. An automatization of the segmentation process would lead to a more time-efficient analysis. In the present work, the manually segmented thigh magnetic resonance imaging database MyoSegmenTUM is presented. It hosts water-fat MR images of both thighs of 15 healthy subjects and 4 patients with NMD with a voxel size of 3.2x2x4 mm^3^ with the corresponding segmentation masks for four functional muscle groups: quadriceps femoris, sartorius, gracilis, hamstrings. The database is freely accessible online at https://osf.io/svwa7/?view_only=c2c980c17b3a40fca35d088a3cdd83e2. The database is mainly meant as ground truth which can be used as training and test dataset for automatic muscle segmentation algorithms. The segmentation allows extraction of muscle cross sectional area (CSA) and volume. Proton density fat fraction (PDFF) of the defined muscle groups from the corresponding images and quadriceps muscle strength measurements/neurological muscle strength rating can be used for benchmarking purposes.

## Introduction

The non-invasive evaluation of muscle tissue with magnetic resonance imaging (MRI) has recently gained a lot of interest to assess muscle strength, neuromuscular diseases (NMD), musculoskeletal disorders, metabolic diseases and aging effects. An increased signal on T2-weighted MR images is observed after exercise [1–5] and influence of training effects have been shown in MR images [6, 7]. In NMD the two main characteristic pathologies are acute edematous muscle alterations and fatty infiltration of chronically affected muscles [8–10]. In other musculoskeletal disorders such as osteoarthritis muscle proton density fat fraction (PDFF) has been related to symptomatic and structural severity [11]. Regional distribution of intramuscular adipose tissue has also been shown to be different in patients with metabolic diseases such as type 2 diabetes [12].

Conventional magnetic resonance imaging of muscles includes T1-weighted and T2-weighted Short Tau Inversion Recovery (STIR) sequences. Based on such images a qualitative assessment of the described changes can be performed and semi-quantitative rating scales for changes in the muscles tissue can be applied [13–16]. However, the hereby performed analysis is highly dependent on the subjective judgment of the reader and a longitudinal evaluation of training effects or disease progression remains challenging. To allow a more objective analysis there is an emerging need for quantitative MR data.

Muscle hypertrophy or atrophy can be accessed by the cross sectional area (CSA) of the muscle. The CSA can be calculated based on anatomical images of the musculature. Using quantitative chemical shift encoding-based water-fat MRI fatty infiltration in muscle tissue can be additionally evaluated [17–19].

PDFF and CSA of muscle groups and individual muscles can be related to biometrically measured muscle strength [20–22]. In patients with NMD characteristic patterns of fatty infiltration especially in the thigh have been defined [23] representing a promising approach for diagnosis of the underlying disease using MR images.

For the subsequent analysis of the quantitative MR data that allows the investigation of single muscle groups, regions of interest (ROIs) have to be defined through delineation of the muscle contours [24]. Despite recent advances on semi-automatic or automatic segmentation methods, their application in clinical settings is still difficult [25], and the segmentation of the muscle often needs to be performed mainly manually for every single muscle or muscle group. Skeletal muscle manual segmentation is a very time consuming process and a bottleneck in the widespread clinical application of quantitative skeletal muscle MRI. A reliable, robust and fast automated or semi-automated way of muscle segmentation would be highly beneficial for the analysis of quantitative muscle MR images. An automatic muscle segmentation would also improve multi-parametric analysis of different biomarkers because the defined ROIs could be easily applied on other sequences covering the same anatomical region. Already existing methods would highly benefit from further evaluation and have yet been only applied on a ground truth of healthy subjects to the best of our knowledge [25–29].

Particularly the thigh is a region of high interest for quantitative muscle MRI because of its technical, anatomical and functional advantages. The thigh is a region of good magnetic field homogeneity with relatively low motion artefacts. Patients with NMD show the disease characteristic patterns mainly in the thigh and lower leg [23] and it is possible to perform muscle selective exercise in this region.

In the present work, a database offering free online access to manually segmented thigh muscles of healthy volunteers and NMD patients is reported. A ground truth MR image database is introduced aiming to facilitate access to manually segmented images and to become an essential tool as training or test dataset in developing automatic segmentation methods for thigh muscles. Volumetric information for the segmented muscle groups as well as strength measurements are also provided.

## Materials and methods

### Subjects

The study was approved by the institutional Committee for Human Research (Ethikkommission der Fakultaet fuer Medizin der TU Muenchen). All subjects gave written informed consent before participation in the study.

The database contains 21 MRI datasets of healthy volunteers (males n = 12, female n = 3, 29.1 +/- 7.7 years) (3 subjects were scanned 3 times with repositioning for reproducibility measurements) and 4 datasets of patients with different neuromuscular diseases: myotonic dystrophy type 2 (DM2) (n = 2), limb girdle muscular dystrophy (LGMD) 2A (n = 1), amyotrophic lateral sclerosis (ALS) (n = 1) (male n = 2, female n = 2, 52.8 +/- 8.9 years).

### MR imaging

The bilateral thigh muscles were scanned on a 3 Tesla system (Philips, Ingenia, Best, Netherlands). Scanning was performed in 2 (healthy volunteers) or 3 (patients) consecutive axial stacks to cover bilaterally the whole thigh from the hip down to the cranial edge of the patella. The built-in 12-channel posterior coil and a 16-channel anterior coil were used, which was placed on top of the hip and thigh region to ensure best signal quality for the scanned muscle groups.

For the healthy volunteers HV001 to HV011 a six-echo 3D spoiled gradient echo sequence was used for chemical shift encoding-based water-fat separation. The sequence acquired the six echoes in a single TR using non-flyback (bipolar) read-out gradients and the following imaging parameters: TR/TE_min_/ΔTE = 10/1.04/0.8 ms, FOV = 300x525 mm^2^, acquisition matrix = 96x263, acquired slice thickness = 4 mm, reconstructed matrix size = 560x560, voxel size = 3.2x2x4 mm^3^, number of slices = 65, receiver bandwidth = 2345 Hz/pixel, frequency direction = A/P (to minimize breathing artifacts), SENSE in L/R direction with reduction factor R = 2, N_avg_ = 1, scan time = 1 min and 48 s per stack. A flip angle of 3° was used to minimize T_1_-bias effects.

For the healthy volunteers HV012 to HV015 a six-echo 3D spoiled gradient echo sequence was used for chemical shift encoding-based water-fat separation. The sequence acquired the six echoes in a single TR using non-flyback (bipolar) read-out gradients and the following imaging parameters: TR/TE_min_/ΔTE = 6.4/1.1/0.8 ms, FOV = 220x400 mm^2^, acquisition matrix = 68x150, acquired slice thickness = 4 mm, reconstructed matrix size = 432x432, voxel size = 3.2x2.2x4 mm^3^, number of slices = 63, receiver bandwidth = 2484 Hz/pixel, frequency direction = A/P (to minimize breathing artifacts), N_avg_ = 1, scan time = 1 min and 25 s per stack. A flip angle of 3° was used to minimize T_1_-bias effects.

For the patients, a six-echo 3D spoiled gradient echo sequence was used for chemical shift encoding-based water-fat separation. The sequence acquired the six echoes in a single TR using non-flyback (bipolar) read-out gradients and the following imaging parameters: TR/TE_min_/ΔTE = 10/1.04/0.8 ms, FOV = 262x424 mm^2^, acquisition matrix = 84x211, acquired slice thickness = 8 mm, reconstructed matrix size = 512x512, voxel size = 3.2x2x4 mm^3^, number of slices = 30, receiver bandwidth = 2325 Hz/pixel, frequency direction = A/P (to minimize breathing artifacts), SENSE in L/R direction with reduction factor R = 2, N_avg_ = 1, scan time = 20 s per stack. A flip angle of 3° was used to minimize T_1_-bias effects.

The gradient echo imaging data were processed online using the multi-echo mDIXON fat quantification method provided by the manufacturer. Specifically, a complex-based water-fat decomposition was performed using a single T_2_* correction and a pre-calibrated fat spectrum, accounting for the presence of the multiple peaks in the fat spectrum. A seven-peak fat spectrum model was employed. The imaging-based proton density fat fraction (PDFF) map was computed as the ratio of the fat signal over the sum of fat and water signals.

Axial water images, axial fat images and axial PDFF maps of each stack were stored as separate datasets for each subject as a *.dcm file and a *.nii file (https://nifti.nimh.nih.gov/nifti-1).

### MR image segmentation

Muscle segmentation was performed by manually drawing regions of interest (ROIs) on the PDFF maps using the open access image viewer software MITK (German Cancer Research Center, Division of Medical and Biological Informatics, Medical Imaging Interaction Toolkit, Heidelberg, Germany). The ROIs delineated the following clinically relevant muscle groups: quadriceps femoris muscle, sartorius muscle, gracilis muscle and hamstring muscles. The ROIs were placed in each muscle group with a margin of approximately 2 mm to their outer contour to avoid the accidental inclusion of subcutaneous fat and the muscle fat-interface as previously reported [20]. The ROIs extend from the cranial beginning of the muscle groups down to the muscle tendon transition at the knee. The segmentations were performed by one operator (2 years of experience in imaging of NMD patients) in the images of all 19 subjects including those acquired for reproducibility purposes and reviewed by a board certified radiologist (10 years of experience in musculoskeletal radiology). The average segmentation time was approximately 6 hours for one subject. The manual segmentation of each muscle group is available as a binary mask, in which pixels with intensity value of 1 correspond to muscle tissue, while pixels with value 0 to the background. Each mask of each image stack was stored as a separate *.mha file. Consequently, each subject dataset has 16 to 24 corresponding segmentation masks including both legs and all stacks.

### Muscle strength measurements/Neurological muscle strength rating

Right quadriceps muscle maximum isometric torque [Nm] produced by knee extension at 60° and 90° knee flexion angle was obtained in healthy volunteers (HV001 to HV011) by using a rotational dynamometer (Isomed 2000, D&R Fertsl GmbH, Hemau, Germany). In HV012 to HV15 isometric muscle strength measurements were performed bilaterally at 60°. The subjects were seated in upright position (90° hip flexion) and carefully fastened with safety belts to avoid any kind of additional movement. The aim was to generate the individual maximum isometric torque in the quadriceps muscle at 60° and 90° knee flexion angle. The subjects performed three repetitions with maximum isometric muscle activity by full recovery in between and the highest value in each angle was used for the data analysis as previously reported [20].

Neurological muscle strength rating was performed by a board certified neurologist bilaterally in the thigh of all 4 patients for knee flexion and knee extension using the Medical Research Council (MRC) score [30]: 0/5 no contraction; 1/5 muscle flicker, but no movement; 2/5 movement possible, but not against gravity; 3/5 movement possible against gravity, but not against resistance by the examiner; 4/5 movement possible against some resistance by the examiner; 5/5 normal strength.

## Results

The manually segmented thigh magnetic resonance imaging database is available online at https://osf.io/svwa7/?view_only=c2c980c17b3a40fca35d088a3cdd83e2. It includes gender, age, weight and height for each subject and quadriceps muscle strength measurements of the healthy volunteers as well as the neurological muscle strength rating (MRC score) of the patients (Tables 1 and 2). Axial water images, axial fat images and axial PDFF maps of each stack were deposited as separate datasets for each subject as *.dcm file and *.nii file. The segmentation masks of the four muscle groups of both legs in all stacks were deposited as *.mha files.

**Table 1:** Gender, age, weight, height, quantitative muscle strength measurements of healthy volunteers.

SUBJECT ID	GENDER	AGE	WEIGHT [KG]	HEIGHT [CM]	R_QF_STRENGTH MEASUREMENT 60° [NM]	R_QF_STRENGTH MEASUREMENT 90° [NM]	L_QF_STRENGTH MEASUREMENT 60° [NM]
**HV001**	m	30	99	179	369	323	
**HV002**	m	25	103	191	372	231	
**HV003**	m	21	96	186	336	270	
**HV004**	m	32	88	177	276	204	
**HV005**	m	48	89	192	245	183	
**HV006**	m	26	90	180	321	204	
**HV007**	m	22	64	174	246	157	
**HV008**	m	25	86	189	316	225	
**HV009**	m	27	85	182	282	224	
**HV010**	f	24	69	169	220	120	
**HV011**	m	20	115	177	218	202	
**HV012**	f	39	72	164	210		159
**HV013**	f	25	76	170	176		187
**HV014**	m	31	87	189	233		216
**HV015**	m	41	77	174	237		228

**Table 2:** Gender, age, weight, height, MRC rating of muscle strength of patients.

SUBJECT ID	GENDER	AGE	WEIGHT [KG]	HEIGHT [CM]	DISEASE	MRCSCORE_R_KNEE_FLEXION	MRCSCORE_L_KNEE_FLEXION	MRCSCORE_R_KNEE_EXTENSION	MRCSCORE_L_KNEE_EXTENSION
**P001**	m	52	95	181	DM2	5/5	5/5	5/5	5/5
**P002**	f	52	79	168	LGMD2A	4/5	4/5	5/5	5/5
**P003**	m	41	98	191	ALS	5/5	5/5	4/5	5-/5
**P004**	f	66	90	165	DM2	5/5	5/5	5/5	5/5

**Fig 1:**
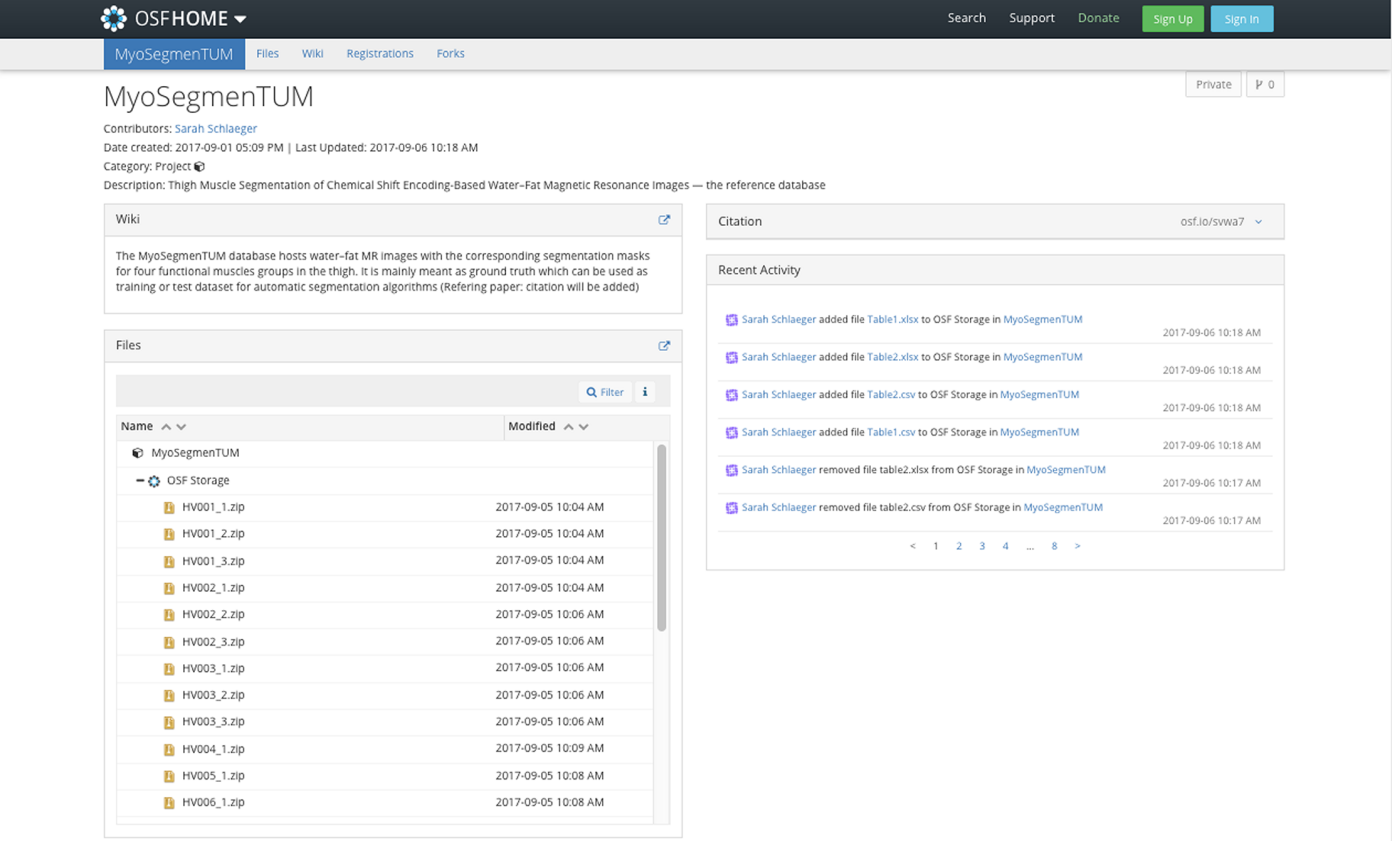
Interface of the MyoSegmenTUM database.

Fig 1 shows the interface of the MyoSegmenTUM database. Datasets of subjects and corresponding segmentation masks are labeled with the same subject ID (HV for healthy volunteer, P for patient) and stack number. Masks were marked left (L) or right (R) and named after the muscle group: QF (quadriceps femoris muscle), SA (sartorius muscle), GR (gracilis muscle), HS (hamstring muscles).

**Fig 2:**
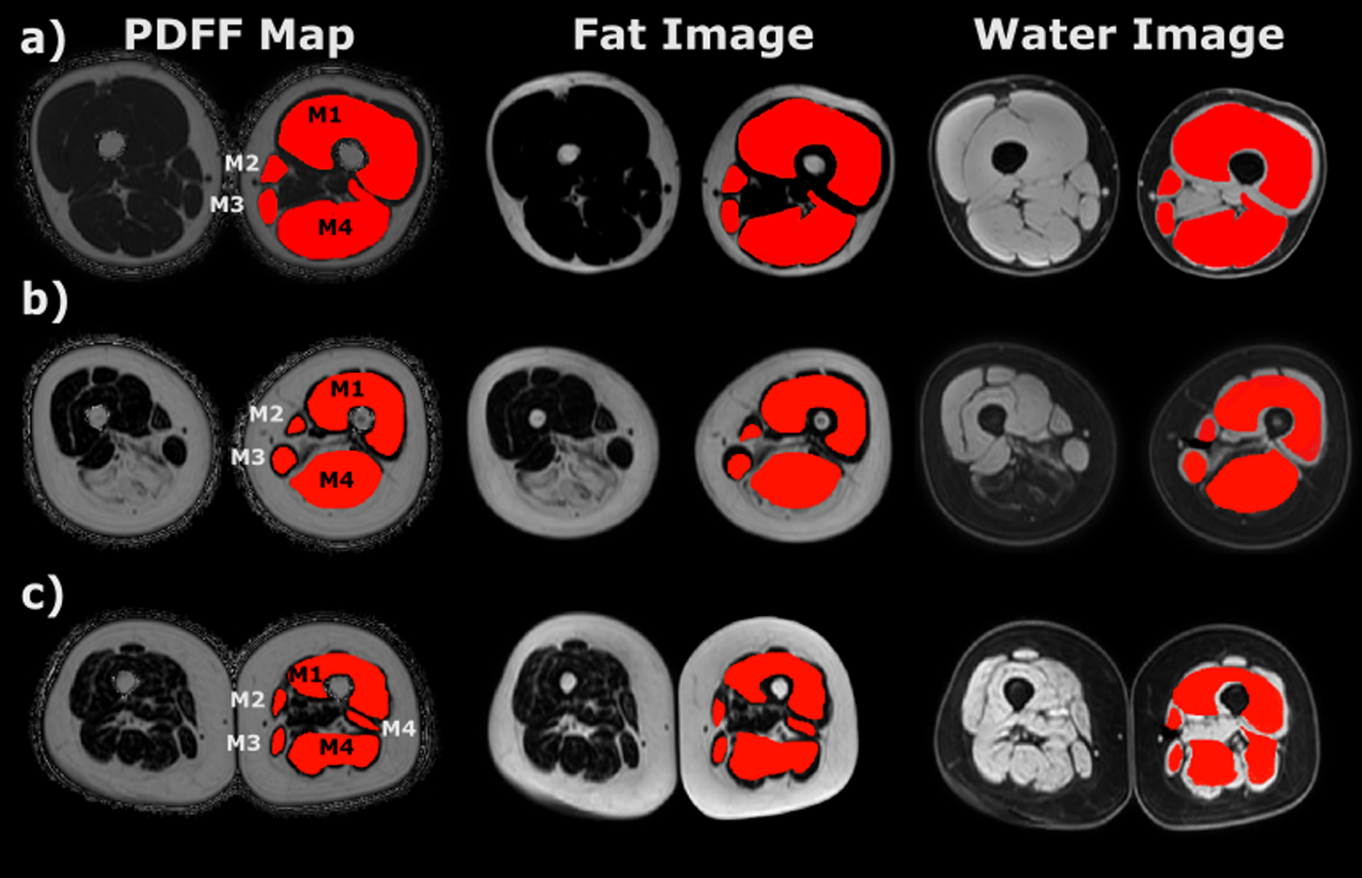
Water Images, Fat Images, PDFF Maps of Healthy Volunteer (a) / LGMD2A Patient (b) / DM2 Patient (c) with Segmentation Masks on the Left Leg.

Fig 2 shows the water images (muscle bright), fat images (fat bright) and PDFF maps of a healthy volunteer (a) contrasted to the images of patients with LGMD2A (b) and DM2 (c) showing the characteristic pathological involvement patterns of these diseases. On the left leg, the four different muscle segmentation masks are highlighted, respectively.

In Tables 3 to 6 the PDFF and the volume of all muscle groups of all healthy volunteers and patients are summarized. Each table contains the values for one muscle group bilaterally, respectively.

**Table 3:** PDFF and muscle volume of healthy volunteers and patients in quadriceps femoris muscle.

Subject ID	R_QF_PDFF_mean	R_QF_PDFF_SD	R_QF_V [mm^3^]	L_QF_PDFF_mean	L_QF_PDFF_SD	L_QF_V [mm^3^]
**HV001_1**						
stack1	3.67	4.77	1718788.00	3.58	4.15	1658052.00
stack2	3.55	4.40	1386032.00	3.88	4.82	1421528.00
**HV001_2**						
stack1	3.19	4.45	1692708.00	3.26	3.96	1747708.00
stack2	3.52	4.60	1449972.00	3.70	4.45	1334548.00
**HV001_3**						
stack1	3.02	4.14	1709228.00	3.57	3.90	1727000.00
stack2	3.53	4.04	1389408.00	3.84	4.42	1331776.00
**HV002_1**						
stack1	3.81	4.62	1954700.00	4.38	4.59	2036844.00
stack2	4.72	6.24	1175412.00	4.95	5.29	1256168.00
**HV002_2**						
stack1	3.78	4.68	2024556.00	4.35	4.62	1918668.00
stack2	4.24	4.88	1261884.00	4.79	5.28	1381964.00
**HV002_3**						
stack1	3.89	4.33	2010636.00	4.19	4.55	1938084.00
stack2	4.45	4.41	1250028.00	4.84	5.24	1313176.00
**HV003_1**						
stack1	3.17	4.03	1496736.00	3.71	4.98	1659248.00
stack2	4.34	6.56	977720.00	4.32	6.24	961172.00
**HV003_2**						
stack1	3.63	4.09	1645324.00	3.53	4.89	1673008.00
stack2	4.44	5.68	978748.00	4.46	5.93	978240.00
**HV003_3**						
stack1	3.34	3.96	1640932.00	3.42	4.91	1670328.00
stack2	4.24	5.70	994680.00	4.28	5.90	991892.00
**HV004_1**						
stack1	6.44	7.95	1326784.00	7.04	8.39	1481804.00
stack2	7.45	9.07	889560.00	7.03	8.61	969892.00
**HV005_1**						
stack1	6.12	6.68	1449180.00	5.77	6.09	1432808.00
stack2	7.50	7.47	929148.00	7.34	7.79	953472.00
**HV006_1**						
stack1	3.23	4.14	1275444.00	3.13	3.63	1359984.00
stack2	4.11	6.10	1210860.00	4.04	5.81	1282804.00
**HV007_1**						
stack1	2.67	3.45	1241308.00	2.48	2.97	1313296.00
stack2	3.03	3.31	876612.00	3.04	3.55	1009576.00
**HV008_1**						
stack1	3.67	4.00	1526608.00	3.43	3.75	1736964.00
stack2	4.12	4.54	767012.00	4.30	4.52	867820.00
**HV009_1**						
stack1	3.15	4.55	1643956.00	3.51	5.01	1898412.00
stack2	4.89	7.27	1145252.00	4.72	5.89	1260916.00
**HV010_1**						
stack1	3.08	3.66	812500.00	3.76	4.04	766984.00
stack2	4.70	6.97	951556.00	4.36	4.82	1059224.00
**HV011_1**						
stack1	3.21	3.95	861920.00	3.77	4.10	771444.00
stack2	4.74	7.03	984340.00	4.43	5.12	1091804.00
**HV012_1**						
stack1	3.29	9.02	236376.00	3.00	5.40	254560.00
stack2	3.69	7.09	830744.00	3.90	5.97	775104.00
**HV013_1**						
stack1	1.34	4.37	542664.00	1.74	3.53	480972.00
stack2	2.53	4.88	1070572.00	3.14	4.17	950440.00
**HV014_1**						
stack1	1.28	4.04	699392.00	1.89	3.49	801772.00
stack2	0.81	3.28	941420.00	1.88	3.77	976860.00
**HV015_1**						
stack1	1.50	3.47	595036.00	2.09	3.39	568980.00
stack2	0.73	3.60	880296.00	2.07	3.74	796276.00
**P001_1**						
stack1	13.05	10.62	241114.00	10.88	9.71	261194.00
stack2	12.58	12.35	232985.00	12.87	13.65	235698.00
stack3	15.67	14.89	81634.00	16.45	15.99	81017.00
**P002_1**						
stack1	8.22	7.20	77556.00	9.01	9.08	71992.00
stack2	8.99	10.21	176872.00	9.47	11.16	175013.00
stack3	11.04	11.24	94434.00	11.94	12.83	97685.00
**P003_1**						
stack1	14.45	10.94	104634.00	10.15	9.03	138679.00
stack2	14.34	10.82	180092.00	9.40	8.43	224038.00
stack3	16.09	13.05	119786.00	11.46	10.64	145572.00
**P004_1**						
stack1	15.67	14.77	130906.00	16.90	15.18	116857.00
stack2	16.89	15.04	136849.00	16.72	15.00	125830.00
stack3	20.53	14.37	14227.00	19.66	12.48	12167.00
**HV_mean**	**3.71**		**1201096.00**	**3.93**		**1234799.33**
**P_mean**	**13.96**		**132590.75**	**12.91**		**140478.50**

**Table 4:** PDFF and muscle volume of healthy volunteers and patients in sartorius anterior muscle.

Subject ID	R_SA_PDFF_mean	R_SA_PDFF_SD	R_SA_V [mm^3^]	L_SA_PDFF_mean	L_SA_PDFF_SD	L_SA_V [mm^3^]
**HV001_1**						
stack1	5.79	4.57	102692.00	4.50	3.91	100072.00
stack2	5.38	4.71	73076.00	4.11	4.48	81932.00
**HV001_2**						
stack1	5.50	4.91	112892.00	5.08	4.34	113844.00
stack2	4.94	4.48	87256.00	4.87	5.81	93872.00
**HV001_3**						
stack1	5.12	4.65	126308.00	4.28	4.09	120360.00
stack2	4.49	4.76	91392.00	4.53	4.83	87812.00
**HV002_1**						
stack1	6.36	4.48	164728.00	6.09	4.42	173492.00
stack2	5.63	5.92	93796.00	4.98	5.15	91872.00
**HV002_2**						
stack1	5.75	4.50	136116.00	5.18	4.22	164504.00
stack2	5.44	5.41	95924.00	4.92	5.44	101076.00
**HV002_3**						
stack1	5.47	4.45	141620.00	5.01	4.28	172592.00
stack2	5.96	5.59	87308.00	5.63	5.83	97816.00
**HV003_1**						
stack1	3.40	4.27	142852.00	4.28	3.96	140016.00
stack2	4.07	4.55	93552.00	4.72	5.98	96012.00
**HV003_2**						
stack1	5.43	3.69	130808.00	4.24	4.11	142556.00
stack2	4.50	4.24	93600.00	5.33	6.31	96092.00
**HV003_3**						
stack1	4.31	3.72	116132.00	4.24	4.00	135420.00
stack2	4.80	4.06	84312.00	5.42	6.53	101488.00
**HV004_1**						
stack1	9.59	7.30	110676.00	8.73	6.10	110380.00
stack2	9.74	7.85	78236.00	8.35	8.27	85544.00
**HV005_1**						
stack1	8.21	5.38	92580.00	7.69	5.16	118348.00
stack2	9.29	6.70	68256.00	10.19	7.09	82760.00
**HV006_1**						
stack1	5.77	5.30	102672.00	6.62	6.23	129392.00
stack2	6.34	6.26	80556.00	6.30	6.74	93116.00
**HV007_1**						
stack1	4.75	3.94	84816.00	5.30	3.73	82656.00
stack2	4.94	3.57	58608.00	5.27	4.43	55140.00
**HV008_1**						
stack1	4.59	4.08	103544.00	5.62	4.21	109360.00
stack2	4.55	3.89	65068.00	4.77	5.11	66268.00
**HV009_1**						
stack1	5.92	5.05	130248.00	6.55	5.48	143068.00
stack2	6.96	6.21	92024.00	6.70	6.18	95740.00
**HV010_1**						
stack1	8.05	6.16	40516.00	7.77	6.60	47068.00
stack2	6.88	6.77	36280.00	9.48	8.87	46612.00
**HV011_1**						
stack1	8.09	6.10	40072.00	8.64	7.45	52752.00
stack2	6.97	6.69	37136.00	9.28	8.78	46040.00
**HV012_1**						
stack1	3.64	4.95	21704.00	3.78	4.68	19944.00
stack2	5.40	5.05	24788.00	5.10	5.20	26960.00
**HV013_1**						
stack1	3.49	5.21	27632.00	4.09	5.27	24908.00
stack2	3.58	5.40	32056.00	4.02	4.79	27072.00
**HV014_1**						
stack1	3.95	4.21	18060.00	4.88	3.90	25272.00
stack2	1.19	4.08	6708.00	2.69	5.05	22720.00
**HV015_1**						
stack1	3.07	4.49	34488.00	3.91	4.36	33900.00
stack2	2.27	4.41	38368.00	3.95	4.25	30072.00
**P001_1**						
stack1	11.63	6.21	14978.00	15.29	18.26	21032.00
stack2	10.55	8.38	17106.00	11.12	9.31	17797.00
stack3	9.14	6.39	11508.00	9.88	7.57	12504.00
**P002_1**						
stack1	10.16	5.81	6689.00	10.98	7.04	8499.00
stack2	13.58	7.38	6519.00	13.72	7.90	7411.00
stack3	12.15	7.57	3959.00	10.68	7.51	5141.00
**P003_1**						
stack1	12.38	5.18	7023.00	10.83	6.61	8220.00
stack2	12.90	7.04	9478.00	10.84	6.78	8643.00
stack3	12.62	7.83	8289.00	8.90	5.99	8514.00
**P004_1**						
stack1	17.18	8.19	9686.00	16.86	9.67	9063.00
stack2	18.78	9.09	7873.00	16.26	8.36	8554.00
stack3	22.04	11.09	4438.00	20.86	9.71	4965.00
**HV_mean**	**5.47**		**80939.43**	**5.65**		**87760.00**
**P_mean**	**13.59**		**8962.17**	**13.02**		**10028.58**

**Table 5:** PDFF and muscle volume of healthy volunteers and patients in gracilis muscle.

Subject ID	R_GR_PDFF_mean	R_GR_PDFF_SD	R_GR_V [mm^3^]	L_GR_PDFF_mean	L_GR_PDFF_SD	L_GR_V [mm^3^]
**HV001_1**						
stack1	4.79	3.96	63808.00	5.41	5.01	54992.00
stack2	4.42	4.66	63692.00	4.83	5.13	59784.00
**HV001_2**						
stack1	4.11	4.80	64692.00	4.28	4.73	55536.00
stack2	4.42	4.57	58832.00	4.88	5.81	60684.00
**HV001_3**						
stack1	4.38	4.53	69516.00	5.15	5.22	58480.00
stack2	3.97	5.18	65648.00	4.21	5.09	54112.00
**HV002_1**						
stack1	3.88	5.02	105060.00	3.98	4.95	90952.00
stack2	3.44	4.10	58040.00	4.46	6.10	63008.00
**HV002_2**						
stack1	4.17	5.31	99604.00	4.28	4.82	90516.00
stack2	3.50	4.38	72856.00	4.15	5.24	67384.00
**HV002_3**						
stack1	4.48	4.41	91508.00	4.56	4.79	96432.00
stack2	3.76	4.68	65404.00	4.44	4.74	61092.00
**HV003_1**						
stack1	2.98	3.12	104428.00	3.40	3.70	110552.00
stack2	2.18	3.50	75792.00	2.36	3.74	75236.00
**HV003_2**						
stack1	2.60	3.51	111120.00	2.55	3.69	108688.00
stack2	2.86	3.36	76616.00	2.58	3.97	75044.00
**HV003_3**						
stack1	3.23	3.44	110032.00	3.57	3.67	109160.00
stack2	3.21	3.57	79572.00	2.93	3.87	78820.00
**HV004_1**						
stack1	6.67	6.09	55588.00	6.43	6.07	46232.00
stack2	7.30	6.72	33924.00	7.14	7.05	33552.00
**HV005_1**						
stack1	5.72	4.84	75540.00	5.62	4.59	91100.00
stack2	6.64	5.22	49572.00	6.92	5.33	65604.00
**HV006_1**						
stack1	5.03	5.04	76920.00	5.53	5.31	74204.00
stack2	5.59	5.28	77692.00	6.32	6.32	73620.00
**HV007_1**						
stack1	2.59	3.44	105132.00	2.38	3.52	69276.00
stack2	3.10	3.39	71068.00	3.46	3.88	50096.00
**HV008_1**						
stack1	3.08	4.05	94616.00	3.38	4.11	92760.00
stack2	3.61	4.34	46052.00	3.92	5.29	46128.00
**HV009_1**						
stack1	3.63	5.38	64984.00	3.76	4.66	64652.00
stack2	5.60	5.57	45128.00	5.36	5.25	45420.00
**HV010_1**						
stack1	5.81	5.72	36648.00	5.50	5.59	33008.00
stack2	3.45	6.08	43788.00	4.23	7.91	46544.00
**HV011_1**						
stack1	5.96	6.17	37448.00	7.70	9.32	40128.00
stack2	4.04	6.94	44280.00	4.53	8.46	47088.00
**HV012_1**						
stack1	3.64	10.44	4016.00	-0.91	4.51	7584.00
stack2	6.47	5.85	12428.00	2.35	4.27	37980.00
**HV013_1**						
stack1	0.18	5.38	18224.00	1.79	4.92	23872.00
stack2	2.98	4.73	36456.00	2.88	4.56	40472.00
**HV014_1**						
stack1	1.31	4.73	16360.00	2.35	4.74	15204.00
stack2	0.85	4.00	21856.00	1.88	3.83	16288.00
**HV015_1**						
stack1	2.64	3.90	18836.00	4.29	4.14	14728.00
stack2	1.31	4.37	37580.00	2.33	3.48	23324.00
**P001_1**						
stack1	8.89	5.30	13575.00	9.36	5.23	14346.00
stack2	7.43	5.73	15209.00	7.34	5.21	17009.00
stack3	9.08	7.47	2317.00	7.44	6.14	3006.00
**P002_1**						
stack1	5.15	3.52	3118.00	4.36	3.47	4499.00
stack2	6.40	4.59	15801.00	6.87	5.17	21550.00
stack3	6.67	7.32	4272.00	5.44	6.34	5942.00
**P003_1**						
stack1	7.64	4.91	4177.00	6.79	5.30	5421.00
stack2	7.18	4.97	8308.00	6.49	4.76	10227.00
stack3	9.40	7.39	3947.00	7.37	5.70	3830.00
**P004_1**						
stack1	9.66	5.97	9527.00	10.38	5.96	8217.00
stack2	11.36	7.11	7976.00	12.74	9.29	8224.00
stack3	17.81	10.21	69.00	16.35	8.99	65.00
**HV_mean**	**3.89**		**59149.06**	**4.08**		**58793.71**
**P_mean**	**8.89**		**7358.00**	**8.41**		**8528.00**

**Table 6:** PDFF and muscle volume of healthy volunteers and patients in hamstring muscles.

Subject ID	R_HS_PDFF_mean	R_HS_PDFF_SD	R_HS_V [mm^3^]	L_HS_PDFF_mean	L_HS_PDFF_SD	L_HS_V [mm^3^]
**HV001_1**						
stack1	3.07	6.07	176260.00	2.91	5.95	205804.00
stack2	4.84	7.68	666412.00	4.13	6.67	717328.00
**HV001_2**						
stack1	1.81	5.79	182504.00	2.16	5.56	240724.00
stack2	4.74	7.52	697460.00	4.05	6.59	701600.00
**HV001_3**						
stack1	1.34	4.99	203704.00	1.98	5.50	253208.00
stack2	4.23	5.60	683032.00	4.04	6.33	687792.00
**HV002_1**						
stack1	3.44	6.49	408164.00	3.84	6.65	456140.00
stack2	5.77	7.44	671628.00	5.25	5.93	742308.00
**HV002_2**						
stack1	3.47	5.75	395988.00	4.11	6.05	385528.00
stack2	4.96	5.65	804140.00	5.09	6.19	759040.00
**HV002_3**						
stack1	3.91	5.69	441320.00	4.41	6.47	433824.00
stack2	5.46	5.72	766964.00	5.50	5.72	755320.00
**HV003_1**						
stack1	2.28	4.68	373952.00	1.25	4.76	452760.00
stack2	3.47	5.50	590428.00	2.91	4.89	667224.00
**HV003_2**						
stack1	1.24	5.40	466012.00	1.04	4.65	459748.00
stack2	3.39	4.64	655964.00	2.67	4.63	673252.00
**HV003_3**						
stack1	1.70	5.16	427964.00	1.42	4.74	440772.00
stack2	3.31	4.52	681852.00	2.57	4.63	691320.00
**HV004_1**						
stack1	7.72	9.13	263464.00	7.81	9.57	268544.00
stack2	8.70	8.73	454440.00	8.96	8.81	428556.00
**HV005_1**						
stack1	8.12	8.93	363568.00	8.31	8.53	398280.00
stack2	10.83	9.10	753024.00	11.37	9.34	769420.00
**HV006_1**						
stack1	3.91	6.68	264332.00	4.58	7.02	261692.00
stack2	3.80	5.28	820648.00	3.94	6.16	783076.00
**HV007_1**						
stack1	0.76	2.75	253508.00	0.68	3.11	257492.00
stack2	2.04	3.27	569920.00	1.82	4.00	557312.00
**HV008_1**						
stack1	1.20	5.35	402488.00	1.67	5.01	479884.00
stack2	2.81	4.30	586336.00	2.64	4.72	594332.00
**HV009_1**						
stack1	3.83	5.63	333972.00	3.44	5.50	335964.00
stack2	5.75	6.11	598464.00	5.23	6.24	557560.00
**HV010_1**						
stack1	3.82	7.06	150660.00	3.63	7.09	139216.00
stack2	3.19	5.17	570516.00	3.50	5.96	584564.00
**HV011_1**						
stack1	4.06	7.41	165604.00	3.58	6.71	142476.00
stack2	3.22	5.28	565832.00	3.40	5.76	562232.00
**HV012_1**						
stack1	5.42	8.53	7564.00	2.04	5.36	21252.00
stack2	6.53	12.97	243104.00	3.54	6.33	241108.00
**HV013_1**						
stack1	1.80	6.98	61120.00	0.79	5.28	60692.00
stack2	4.22	9.49	418704.00	4.81	9.98	384552.00
**HV014_1**						
stack1	0.93	4.74	83836.00	0.39	3.45	83456.00
stack2	1.51	3.82	463164.00	2.21	4.07	435128.00
**HV015_1**						
stack1	1.02	2.83	50380.00	1.43	4.29	48936.00
stack2	0.50	3.50	502792.00	1.57	3.59	502520.00
**P001_1**						
stack1	21.67	14.73	32958.00	15.67	9.38	33655.00
stack2	12.64	14.37	123674.00	11.91	12.28	134167.00
stack3	11.74	10.45	56058.00	12.16	9.09	59976.00
**P002_1**						
stack1	18.67	9.46	4456.00	23.87	15.25	5173.00
stack2	70.29	22.93	60003.00	70.16	23.33	65349.00
stack3	58.67	32.79	80285.00	64.76	28.24	88893.00
**P003_1**						
stack1	5.37	9.21	22135.00	4.51	5.60	23684.00
stack2	8.90	10.12	105870.00	7.42	7.86	105075.00
stack3	14.23	13.60	119446.00	13.44	11.84	116502.00
**P004_1**						
stack1	13.23	11.56	33275.00	13.01	13.34	27916.00
stack2	14.59	12.73	94382.00	15.01	14.40	97686.00
stack3	18.58	13.18	16664.00	16.96	11.33	16550.00
**HV_mean**	**3.76**		**434314.00**	**3.59**		**443379.43**
**P_mean**	**22.38**		**62433.83**	**22.41**		**64552.16667**

## Discussion

In the present work a database for manually segmented thigh muscles in MR images of healthy volunteers and patients with neuromuscular diseases is presented.

The database offers access to axial water, axial fat images and axial PDFF maps of the thigh as well as the corresponding segmentation masks for four functional muscle groups. Therefore, the database offers free access to training or test datasets for automatic segmentation algorithms. Intensity information from the different water and fat contrast can be taken into account in terms of a joint multivariate analysis process to separate muscle and fat voxels [31–33]. Furthermore, the open-access database could be used to benchmark different segmentation methods in a comparable way. The data is available in two (healthy volunteers) and three (NMD patients) consecutive stacks. The stacks were acquired without a gap and consequently could easily be merged by a potential user of the database. However, the separated stacks are uploaded on the database so that every user could chose his region of interest and does not have to download the whole thigh volume if not wanted.

Approximately 80% of the presented images are from healthy volunteers. The healthy muscle tissue and its allocation in the thigh represent the ground truth for automatic segmentation algorithms. The other images represent an insight into typical presentations of three different muscle diseases: DM2, LGMD2A, ALS, being “extreme” cases which are useful to understand the limitations of an applied automatic segmentation method.

The calculated mean PDFF and CSA exemplary illustrate the application of quantitative MRI data in muscle imaging. Tables 3 to 6 can serve as the ground truth when performing benchmark tests with newly developed computer vision or machine learning algorithms and may help to evaluate their performance.

The quantitative data in Tables 3 to 6 can only be extracted after the definition of specific ROIs. As there is an increasing attempt for quantification in muscle MRI to assess acute and chronic changes in the muscle tissue there is a high need for a time efficient way of segmenting the muscle groups or individual muscles. An automatic analysis of quantitative MRI data enables the analysis of big data. This may allow the application of quantitative water-fat imaging in clinical practice resulting in a better monitoring of disease progression and therapy effectiveness. It could foster the development of fully automated diagnostic procedures by computational segmenting and analyzing PDFF maps, followed by an automatic diagnostic process using the characteristic patterns of NMD [23] in axial MR images and thus helping to identify the correct diseases.

The present database offers the possibility to work with whole muscle volumes in the thigh. It is known that quantitative values such as the PDFF and water T2 can differ throughout the muscle volume affecting the muscle heterogeneously along the proximodistal axis [34]. Therefore, a segmentation of the whole muscle volume is essential and offers new insights into muscle physiology and pathology.

The relatively low number of datasets can be seen as a limitation of the present database, particularly in the context of traditional machine learning ground truth databases. However, the database can be extended by more manually segmented muscle imaging data. It is planned to gradually increase the number of datasets by additional datasets of healthy thigh musculature and from patients with various NMD showing less and more extreme pathologies of the muscle tissue. The amount of training data for automatic segmentation algorithms based on neural networks is highly dependent on the desired performance of the segmentation tool and how the available data is altered (mirrored, deformed) to artificially increase the number of training sets. However, the provided database could be seen as a good starting point for the development of automatic algorithms and the planned extension should provide enough datasets for a sufficient training and testing of the segmentation algorithms.

To obtain accurate PDFF results the presented segmentation was placed slightly inside the contour of each muscle group to exclude subcutaneous fat and muscle-fat interface. This might be considered as a second limitation, as an automatic segmentation will have to be performed at the exact muscular border and eroded in a consecutive step for evaluation of PDFF.

## Conclusion

A database (MyoSegmenTUM) for manually segmented thigh muscles in MR images of healthy volunteers and patients with neuromuscular diseases was presented together with the corresponding manual segmentation masks. The database offers training and test datasets for the development of automatic muscle segmentation algorithms which are highly needed to exploit the maximum potential out of quantitative muscle MRI in the future for diagnosis and treatment of muscle pathologies.
